# Variants of MicroRNA Genes: Gender-Specific Associations with Multiple Sclerosis Risk and Severity

**DOI:** 10.3390/ijms160820067

**Published:** 2015-08-24

**Authors:** Ivan Kiselev, Vitalina Bashinskaya, Olga Kulakova, Natalia Baulina, Ekaterina Popova, Alexey Boyko, Olga Favorova

**Affiliations:** 1 Dep. of Molecular Biology and Medical biotechnology, Pirogov Russian National Research Medical University, Moscow 117997, Russia; E-Mails: vitalina.bashinskaya@gmail.com (V.B.); olga.koulakova@gmail.com (O.K.); tati.90@mail.ru (N.B.); olga_favorova@mail.ru (O.F.); 2 Lab. of Functional Genomics of Cardiovascular Diseases, Russian Cardiology Research and Production Complex, Moscow 121552, Russia; 3 Dep. of Neurology, Neurosurgery and Medical Genetics, Pirogov Russian National Research Medical University, Moscow 117997, Russia; E-Mails: epopova1980@yandex.ru (E.P.); boykoan13@gmail.com (A.B.)

**Keywords:** multiple sclerosis, microRNA, SNP, association analysis, susceptibility, MSSS

## Abstract

Multiple sclerosis (MS) is an autoimmune neuro-inflammatory disease arising from complex interactions of genetic, epigenetic, and environmental factors. Variations in genes of some microRNAs—key post-transcriptional regulators of many genes—can influence microRNAs expression/function and contribute to MS via expression changes of protein-coding target mRNA genes. We performed an association study of polymorphous variants of *MIR146A* rs2910164, *MIR196A2* rs11614913, *MIR499A* rs3746444 *MIR223* rs1044165 and their combinations with MS risk and severity. 561 unrelated patients with bout-onset MS and 441 healthy volunteers were enrolled in the study. We observed associations of MS risk with allele MIR223*T and combination (MIR223*T + MIR146A*G/G) carriage in the entire groups and in women at Bonferroni-corrected significance level (*p*_corr_ < 0.05). Besides, MIR146A*G/G association with MS was observed in women with nominal significance (*p*_f_ = 0.025). No MS associations were found in men. A more severe MS course (MSSS value > 3.5) was associated with the carriage of MIR499A*C/T and, less reliably, of MIR499A*C (*p*_corr_ = 0.006 and *p*_corr_ = 0.024, respectively) and with the carriage of combinations (MIR499A*C/T + MIR196A2*C) and (MIR499A*C + MIR196A2*C) (*p*_corr_ = 0.00078 and *p*_corr_ = 0.0059, respectively). These associations also showed gender specificity, as they were not significant in men and substantially reinforced in women. The strongest association with MS severity was observed in women for combination (MIR499A*C/T + MIR196A2*C): *p*_corr_ *=* 4.43 × 10^−6^ and OR = 3.23 (CI: 1.99–5.26).

## 1. Introduction

Multiple sclerosis (MS) is a chronic disease of the central nervous system, characterized by autoimmune inflammation, demyelination, and neurodegeneration [1]. MS usually affects young adults and leads to irreversible disability [2,3]. The more common course, relapsing-remitting MS (RRMS), is characterized by clinical episodes interspersed with periods of stability, affects twice as many women as men and within ten years develops into secondary progressive MS (SPMS) in 40% of patients [4]. Together these two forms are specified as bout-onset MS, which affects approximately 85%–90% of patients.

Clinical phenotypes of the bout-onset MS may vary significantly, including differences in MS progression rate and severity of its clinical manifestations. Several clinical scales have been developed to distinguish individuals with relatively mild and aggressive forms of MS [5]. Among them is the Multiple Sclerosis Severity Score (MSSS) [6], which is acknowledged as the most powerful method for detecting different rates of disease progression [7] and is suitable for different epidemiologic and genetic studies [6]. Early predictors of MS’s course are currently being searched.

MS is a complex disease, the development and clinical phenotypes of which result from interactions between environmental risk factors and genetic susceptibility of polygenic nature. In addition, in the past decade it became clear that epigenetic changes in susceptibility genes, influencing their expression without altering the DNA sequence, may play an essential role in MS development, possibly by mediating the effects of environmental influence [8]. The major mechanisms of epigenetic regulation are DNA methylation and histone modification, which affect the availability of DNA on transcriptional level as well as post-transcriptional gene silencing processes mediated by microRNAs (miRNAs) [9].

MiRNAs are small (19–24 nucleotides long) single-stranded non-coding RNA molecules. In accordance with the latest version of miRBase (release 21), there are more than 1800 annotated human miRNA genes [10]. MiRNAs act in the cell through imperfect base-pairing with target mRNA predominantly in 3′ untranslated region, thereby activating mRNA degradation or repression of its translation [11]. Being conductors of intracellular orchestra, miRNAs are key regulators of many biological processes including immune response [12]. MiRNAs can produce changes in expression of 60% of genes [13], including other miRNA genes, and thus form complicated system similar to the cytokine network, which plays a crucial role in modulation of immune response.

Even a minor shift in miRNA production may lead to significant alterations in gene expression resulting in pathological changes in the immune system. To date, diverse alterations in miRNA biogenesis have been observed in MS and other autoimmune diseases [14,15,16,17]. Differentially expressed miRNAs have been identified in blood elements, cerebrospinal fluid and brain lesions of MS patients [18,19,20].

Previous studies showed a correlation between several polymorphic variants in miRNA genes and their production levels [21,22]. Besides, common single nucleotide polymorphisms (SNPs) in several miRNAs genes were found to be associated with such autoimmune inflammatory diseases as ulcerative colitis, rheumatoid arthritis, and asthma [23,24,25].

Based on the assumption that genetic variation can influence miRNA expression and function, we performed an association study of miRNA gene variants with bout-onset MS risk and severity. The selection of SNPs rs2910164, rs11614913, and rs3746444 in genes *MIR146A*, *MIR196A2*, and *MIR499A*, respectively, was based on evidence for involvement of these miRNAs in inflammatory conditions with autoimmune components [24,25,26]. Importantly, the selected SNPs are located in either pre-miRNAs or mature miRNAs and this may affect their processing and/or function. We also included in the study rs1044165 located near *MIR223* since its association with MS was shown in [27] and needs to be replicated in other populations. Assuming the polygenic nature of MS, we also performed multi-locus association analysis of the studied SNPs with MS susceptibility and severity.

## 2. Results

### 2.1. Analysis of Association of miRNA Genetic Variants with MS Susceptibility

In this study, we used three statistical genetic models: the co-dominant model (effect of heterozygous genotype in comparison with the others); recessive model (effect of homozygous genotype *vs.* the others) and dominant model (effect of allele carriage, *i.e.*, carriage of a given allele as a part of heterozygous or homozygous genotype, *vs.* carriage of the opposite genotype). We considered carriage of any genotype or allele of studied SNPs, which was positively associated with the phenotype, as a risk factor. We also considered as a risk factor carriage of genotype/allele combination if the latter provided a detectable cumulative effect on the phenotype (*i.e.*, *p*-value for this combination was more significant than *p*-values for carriage of individual genotype/allele).

*MIR146A*, *MIR196A2*, and *MIR499A*, and *MIR223* genes are located on the chromosomes 5, 12, and 20, correspondingly. Comparison of allele and genotype carriage rates of *MIR146A* rs2910164, *MIR196A2* rs11614913, and *MIR499A* rs3746444 was performed for the entire groups of Russian MS patients and controls (*i.e.*, without gender stratification) and for women and men separately. Genotype frequencies for these SNPs were in Hardy-Weinberg equilibrium in all groups of patients and healthy individuals and after their stratification by gender, except for frequencies of *MIR146A* SNP in male MS patients (*p* < 0.05). Results of comparison of observed miRNA genotype frequencies with expected frequencies according to the Hardy-Weinberg equilibrium are presented in Table S1.

The *MIR223* gene is located in the X chromosome and classic biallelic genotypes of *MIR223* rs1044165 may be observed only in women. Due to this fact, association analysis for *MIR223* in men and in the entire groups of MS patients and controls was performed for allele carriage only, whereas in women, genotype carriage frequencies were estimated as well. Correspondingly, compliance with Hardy-Weinberg equilibrium was evaluated only for women, and this test was passed successfully (Table S1).

Analysis of carriage of alleles, genotypes and allelic combinations associated with MS susceptibility was performed using the APSampler algorithm. Allele/genotype carriage frequencies for each studied miRNA gene in MS patients and healthy individuals are presented in Table S2. Significant associations of SNPs in *MIR499A* and *MIR196A2* genes with MS risk were not found. Data on genetic variants and their combinations, which were associated with MS with nominal significance at least at one type of comparison (without gender stratification, in women or in men subgroup), are presented in Table 1. Analysis of MS-associated allelic combinations revealed only one biallelic combination, consisting of MIR223*T and MIR146A*G/G, carriage of which was nominally associated with MS at *p*_f_ < 0.01 in the entire group and in women; these data are also included in Table 1.

**Table 1:** Positive associations of SNPs in miRNA genes with MS risk ^†^.

Carriage of Allele/Genotype Combinations	Carriers (%)/Noncarriers (%)		Fisher’s *p*_f_-Value	Bonferroni-Corrected ^‡^ *p*_corr_-Value	OR (95% CI)
-	**Without Gender Stratification**		-		
	**MS Cases, *n* = 561**	**Controls, *n* = 441**			
MIR223*T ^#^	155 (27.6)/406 (72.4)	86 (19.5)/355 (80.5)	**0.0017**	**0.0068**	1.58 (1.17–2.13)
MIR146A*G/G	359 (64.0)/202 (36.0)	268 (60.8)/173 (39.2)	0.16	0.64	1.15 (0.89–1.48)
MIR223*T + MIR146A*G/G	103 (18.4)/458 (81.6)	55 (12.5)/386 (87.5)	**0.0068**	**0.041**	1.58 (1.11–2.25)
-	**Women**		-		
	**MS Cases, *n* = 395**	**Controls, *n* = 285**			
MIR223*T	122 (30.9)/273 (69.1)	65 (22.8)/220 (77.2)	**0.012**	**0.048**	1.51 (1.07–2.15)
MIR146A*G/G	259 (65.6)/136 (34.4)	165 (57.9)/120 (42.1)	**0.025**	0.10	1.39 (1.01–1.90)
MIR223*T + MIR146A*G/G	83 (21.0)/312 (79.0)	37 (13.0)/248 (87.0)	**0.0042**	**0.025**	1.79 (1.17–2.72)
-	**Men**		-		
	**MS Cases, *n* = 166**	**Controls, *n* = 149**			
MIR223*T	33 (19.9)/130 (80.1)	21 (14.1)/128 (85.9)	0.11	0.44	1.51 (0.83–2.75)
MIR146A*G/G	100 (60.3)/66 (39.7)	98 (65.8)/51 (34.2)	0.19	0.76	0.79 (0.50–1.25)
MIR223*T + MIR146A*G/G	20 (12.0)/146 (88.0)	18 (12.1)/131 (87.9)	0.56	1.00	1.00 (0.51–1.97)

^†^ Only data for the SNPs, which were significantly associated with MS at least at one of the comparisons, are presented above. Allele/genotype carriage frequencies for all genes are presented in Table S2; ^‡^ *p*-values were corrected for 4 independent comparisons in case of single alleles/genotypes carriage and for 6 independent comparisons in case of biallelic combinations; ^#^ Hereinafter an allele carriage rate is calculated as a sum of homozygous and heterozygous genotypes containing this allele. Significant *p*_f_ and *p*_corr_-values are in bold; OR—odds ratio; CI—confidential interval.

Carriage of allele MIR223*T by itself was found to be associated with high risk of MS development (*p*_f_ = 0.0017, *p*_corr_ = 0.0068, odds ratio (OR) = 1.58) in the entire group, and this association remained significant in women (*p*_f_ = 0.012, *p*_corr_ = 0.048, OR = 1.51), but not in men after gender stratification. Genotype MIR146A*G/G was enriched in female patients only (*p*_f_ = 0.025, OR = 1.39). Moreover, in women the level of significance increased if both SNPs were included in the biallelic combination (MIR223*T + MIR146A*G/G) (*p*_f_ = 0.0042, *p*_corr_ = 0.025, OR = 1.79). For the entire group this combination was also significantly associated with high MS risk (*p*_f_ = 0.0068, *p*_corr_ = 0.041, OR = 1.58) but the *p*-value was higher than that for MIR223*T separately.

The data above show that MS is associated with MIR223*T and (MIR223*T + MIR146A*G/G) carriage in the entire groups and in women at a Bonferroni-corrected significance level (*p*_corr_ < 0.05). MIR146A*G/G association with MS was observed only in women with nominal significance. For men no significant results were obtained.

### 2.2. Analysis of Association of miRNA Genetic Variants with MS Severity

To estimate MS severity in individual patients, we applied the MSSS value as the most powerful indicator [6]. As shown in Table 2, such clinical features as age at onset, disease duration and mean EDSS value do not differ significantly in female and male MS patients. At the same time, MSSS values were significantly higher in men (Mann-Whitney *p*-value is 0.021). Thus, gender stratification is required in association analysis of MS severity based on MSSS.

**Table 2:** Clinical characteristics of bout-onset MS patients.

Characteristics	MS Patients, *n* = 561	Women, *n* = 395	Men, *n* = 166
Age at onset (years), mean ± SD	27.5 ± 9.2	27.9 ± 9.2	26.41 ± 9.2
Disease duration (years), mean ± SD	11.3 ± 7.4	11.9 ± 7.8	10.1 ± 5.0
EDSS, mean ± SD	2.48 ± 1.17	2.45 ± 1.13	2.54 ± 1.26
MSSS, mean ± SD	3.93 ± 1.97	3.82 ± 1.97 ^†^	4.22 ± 1.96 ^†^
No. of persons with RRMS/SPMS	464/97	329/66	135/31

^†^ According to the Mann-Whitney test, *p* = 0.021. SD—standard deviation; RRMS—relapsing-remitting multiple sclerosis; SPMS—secondary progressive multiple sclerosis.

To search for associations of miRNA gene variants with MS severity, we divided MS patients into two groups with MSSS values ≤3.5 and >3.5. In this way, MS patients with a relatively mild MS course, corresponding to a first three deciles of MSSS score, were compared with patients with medium and severe MS courses [28]. Allele/genotype carriage frequencies for studied miRNA genes in MS patients with MSSS values ≤3.5 and >3.5 are presented in Table S3. Significant after Bonferroni correction association data for carriage of alleles, genotypes and identified biallelic combinations of *MIR499A* and *MIR196A2* variants are presented in Table 3.

Carriage of MIR499A*C allele (*p*_corr_ = 0.00080, odds ratio (OR) = 2.21), especially the MIR499A*C/T genotype (*p*_corr_ = 2.23 × 10^−5^, OR = 2.77), is strongly associated with MS severity in women and, with a lower level of significance, in the entire group (*p*_corr_ = 0.024, OR = 1.61 and *p*_corr_ = 0.0032, OR = 1.85, respectively). Biallelic combinations of these genetic variants with the MIR196A2*C allele are characterized by the essentially higher level of significance and higher OR values. At that, associations for combinations including MIR499A*C/T are more reliable than for those with MIR499A*C and in women they are stronger than without gender stratification. Particularly, for the most significant association of (MIR499A*C/T + MIR196A2*C) with MS severity in women *p*_corr_ is equal to 4.43 × 10^−6^ and OR reached 3.23. At the same time, no significant results were obtained in men. Polymorphic variants of *MIR223* and *MIR146A* genes were associated with MSSS neither in the entire MS group nor upon gender stratification.

**Table 3:** Positive associations of SNPs in miRNA genes with the higher MS severity, evaluated by MSSS value ^†^.

Carriage of Allele/Genotype Combinations	Carriers (%)/Noncarriers (%)		Fisher’s *p*-Value	Bonferroni-Corrected ^‡^ *p*-Value	OR (95% CI)
-	**Without Gender Stratification**		-		
	**MSSS > 3.5, *n* = 304**	**MSSS ≤ 3.5, *n* = 242**			
MIR499A*C/T	112 (36.8)/192 (63.2)	58 (24.0)/184 (76.0)	**0.00081**	**0.0032**	1.85 (1.27–2.70)
MIR499A*C ^#^	119 (39.1)/185 (60.9)	69 (28.5)/173 (71.5)	**0.0059**	**0.024**	1.61 (1.12–2.32)
MIR499A*C/T + MIR196A2*C	101 (33.2)/203 (66.8)	46 (19.0)/196 (81.0)	**0.00013**	**0.00078**	2.12 (1.42–3.16)
MIR499A*C + MIR196A2*C	107 (35.2)/197 (64.8)	55 (22.7)/187 (77.3)	**0.00099**	**0.0059**	1.85 (1.26–2.71)
-	**Women**		-		
	**MSSS > 3.5, *n* = 207**	**MSSS ≤ 3.5, *n* = 181**			
MIR499A*C/T	86 (41.5)/121 (58.5)	37 (20.4)/144 (79.6)	**5.58 × 10^−6^**	**2.23 × 10^−5^**	2.77 (1.76–4.36)
MIR499A*C	89 (43.0)/118 (57.0)	46 (25.4)/135 (74.6)	**0.00020**	**0.00080**	2.21 (1.44–3.41)
MIR499A*C/T + MIR196A2*C	79 (38.2)/128 (61.8)	29 (16.0)/152 (84.0)	**7.38 × 10^−7^**	**4.43 × 10^−6^**	3.23 (1.99–5.26)
MIR499A*C + MIR196A2*C	81 (39.1)/126 (60.9)	37 (20.4)/144 (79.6)	**4.5 × 10^−5^**	**0.00027**	2.50 (1.58–3.95)
-	**Men**		-		
	**MSSS > 3.5, *n* = 97**	**MSSS ≤ 3.5, *n* = 61**			
MIR499A*C/T	26 (26.8)/71 (73.2)	21 (34.4)/40 (65.6)	0.2	0.80	0.70 (0.35–1.40)
MIR499A*C	30 (30.9)/67 (69.1)	23 (37.8)/38 (62.2)	0.24	0.96	0.74 (0.38–1.45)
MIR499A*C/T + MIR196A2*C	7 (7.2)/90 (92.8)	8 (13.1)/53 (86.9)	0.17	1.00	0.52 (0.17–1.50)
MIR499A*C + MIR196A2*C	26 (26.8)/71 (73.2)	18 (29.5)/43 (70.5)	0.42	1.00	0.87 (0.43–1.78)

^†^ Only data for the SNPs, which were significantly associated with MSSS value at least at one of the comparisons, are presented above. Allele/genotype carriage frequencies for all genes are presented in Table S3; ^‡^ *p*-values were corrected using Bonferroni correction for 4 independent comparisons for single alleles/genotypes carriage and for 6 independent comparisons for biallelic combinations; ^#^ Hereinafter an allele carriage rate is calculated as a sum of homozygous and heterozygous genotypes containing this allele. Significant *p*_f_ and *p*_corr_-values are in bold; OR—odds ratio; CI—confidential interval.

We compared also MSSS values in carriers and noncarriers of MIR499A*C/T and MIR499A*C by itself and in combinations with MIR196A2*C. Significant differences in average MSSS values were found for carriers and noncarriers of MIR499A*C/T, MIR499A*C, (MIR499A*C/T + MIR196A2*C) and (MIR499A*C + MIR196A2*C) in the entire group (Mann-Whitney *p*-values are equal to 0.0085, 0.028, 0.0062 and 0.023, respectively). Again, these differences were greater among women (*p* = 0.0004, *p* = 0.0039, *p* = 0.0003, and *p* = 0.0048, respectively) (Table 4). As expected, in men MSSS was not significantly associated with carriage of these variants.

Thus, carriage of the *MIR499A* C/T genotype or C allele by themselves and in combination with MIR196A2*C is strongly associated with high MS severity, estimated by the Multiple Sclerosis Severity Score. This association is women-specific.

**Table 4:** Comparison of MSSS values between carriers and noncarriers of genetic variants associated with MS severity.

Group of Patients	Carriers		Noncarriers			Mann-Whitney *p*-Value
	No. of Persons (%)	MSSS, Mean ± SD	No. of Persons (%)		MSSS, Mean ± SD	
MIR499A*C/T							
All persons	170 (31.1)	4.23 ± 1.92	376 (68.9)	3.80 ± 1.98		**0.0085**
Women	123 (31.7)	4.30 ± 1.97	265 (68.3)	3.60 ± 1.93		**0.0004**
Men	47 (29.7)	4.04 ± 1.80	111 (70.3)	4.30 ± 2.03		0.57
MIR499A*C							
All persons	188 (34.4)	4.16 ± 1.93	358 (65.6)		3.81 ± 1.98	**0.028**
Women	135 (34.8)	4.19 ± 1.97	253 (65.2)		3.62 ± 1.94	**0.0039**
Men	53 (33.5)	4.11 ± 1.85	105 (66.5)		4.28 ± 2.02	0.70
MIR499A*C/T + MIR196A2*C							
All persons	147 (26.9)	4.27 ± 1.93	399 (73.1)		3.81 ± 1.97	**0.0062**
Women	108 (27.8)	4.36 ± 1.96	280 (72.2)		3.61 ± 1.93	**0.0003**
Men	39 (24.7)	4.04 ± 1.85	119 (75.3)		4.28 ± 2.00	0.61
MIR499A*C + MIR196A2*C							
All persons	162 (29.7)	4.20 ± 1.96	384 (70.3)		3.82 ± 1.97	**0.023**
Women	118 (30.4)	4.21 ± 1.98	270 (69.6)		3.64 ± 1.94	**0.0048**	
Men	44 (27.8)	4.17 ± 1.91	114 (72.2)		4.24 ± 1.99	0.92	

Significant *p*-values are in bold.

## 3. Discussion

In the present study, we performed an association study of four miRNA genetic variants with MS susceptibility and severity in Russians. We identified the involvement of polymorphous variants of two miRNA genes—*MIR223* and *MIR146A*—in MS susceptibility and of two other miRNA genes—*MIR499A* and *MIR196A2*—in the MS course. In all cases, significant associations were observed in the entire group and in women but not in men, showing gender specificity.

We found that allele MIR223*T carriage is associated with MS susceptibility in Russians, maintaining the Bonferroni correction. Previously, rs1044165 in *MIR223* was shown to be associated with MS in Italians [27], though with the opposite direction of association, and the microRNA was down-regulated in the serum of MS patients [14].

The *MIR223* gene is located on the X chromosome. X chromosome copy number difference among men and women results in difficulties with association studies of SNPs located on it. If the analysis is performed in mixed-gender groups, male SNP genotypes may be set either as homozygous female (resulting in three genotypes in the total sample) or as unique (hemizygous) ones (five genotypes in total). Such approaches are available only if female/male ratios in studied groups are equal, otherwise the resulting associations with SNP genotypes can be confounded by differences in the sex ratio [29]. Search for associations in gender-stratified groups seems to be the most appropriate, though it leads to loss of statistical power because of the decreasing number of individuals. Unfortunately, in the study [27] no data on sex ratio in the control group were provided, and, to our knowledge, gender stratification was not performed. In the present study, to reduce a possible sex bias, we analyzed women and men separately, in addition to analysis of the entire group, to search for gender-specific associations. Only carriage rates of *MIR223* alleles in the entire group and men were compared, while genotype frequencies were considered as well in women separately.

Inconsistency of obtained results with data in an Italian study may be explained also by involvement of different mechanisms to MS pathogenesis in various ethnic groups. rs1044165, being in a linkage disequilibrium (LD) block with *MIR223*, is located within *VSIG4* gene, which acts as a strong inhibitor of T cell activation [30]. Thus we may suggest opposite SNP alleles to influence MS pathogenesis through the effects of different genes in various populations, acknowledging different LD patterns in them.

In addition to *MIR223* by itself, we found an association of biallelic combination (MIR223*T + MIR146A*G/G) with MS susceptibility in women. This combination satisfies the requirement of the minimal set (combination) of alleles as a genetic risk factor, which means that a combination is associated with the phenotype more reliably (e.g., with better *p*-value) than each of its components [31]. Meanwhile, the same combination cannot be considered a minimal set in the entire group, though it is nominally significant and withstands the Bonferroni correction, since the significance level of association of MIR223*T by itself (*p*_corr_ = 0.0068) is lower than that of the combination (*p*_corr_ = 0.041). Besides, MIR146A*G/G by itself was nominally associated with MS in women (uncorrected *p*_f_-value = 0.025), so the role of *MIR146A* in MS seems to be clarified with a larger sample size and in populations of other descent.

rs2910164 is located in *pre-miR-146a*, and G allele carriage leads to increased mature miR-146a production due to more effective *pri-miR-146a* processing in nucleus and more effective nuclear pore complex binding [22]. Results of recent MS association studies of *MIR146A* gene polymorphism are conflicting. Association of *MIR146A* rs2910164 with MS was not observed in Italians [14], whereas the C allele at this SNP was significantly enriched in Chinese female patients [32]. Such contradictory results can be at least partially explained by large discrepancies in allele frequencies in these populations and even contradictory effects of these alleles on gene expression [32] due to the different evolutionary history of the populations studied to date. In line with emerging data indicating a role of this gene and of the mature miRNA in MS, the same SNP was associated with susceptibility to Behcet’s disease [26], with a clinical phenotype of systemic lupus erythematosus [33] and Alzheimer’s disease [34]. At the same time, no associations were identified for rs2910164 with type 2 diabetes susceptibility in Italians [35] nor with systemic lupus erythematosus or juvenile rheumatoid arthritis in Mexicans [24]. *MIR146A* was suggested to play a role in astrocyte-mediated inflammatory response [36] and to be a part of Th17 pathway [37].

This work also aimed to study the role of miRNA genes as genetic risk factors in MS severity. Finding an adequate measure of this clinical phenotype that enables robust characterization of an individual’s MS course and is applicable to stratification of MS patients in epidemiologic and genetic studies is a serious challenge. Among the first severity criteria was EDSS, a scale which allows the estimation of a neurological patient’s disability at a given period [38]. EDSS as well as other characteristics of current state, e.g., accumulation of brain lesions on MRI, are time-dependent and have to be corrected for disease duration to compare patients [39]. For this purpose, different approaches have been applied in genetic studies—time to reach certain EDSS values [40,41] and the progression index [42,43], that is, the number of EDSS points reached at a given time—but these characteristics do not account for non-linear MS progression. The MSSS value, which relates EDSS to MS duration and compares these characteristics with MS courses in other individuals [6], is probably the optimal tool to measure MS severity, applicable to genetic studies.

We compared allele/genotype/allelic combinations carriage rates of the studied genes in MS patients stratified by their MSSS values. We put the threshold of severe MS course at MSSS value 3.5. This is in accordance with MSSS levels compared in other studies [7,28] and is very close to the median MSSS value in our sample. Analysis was performed separately for male and female patients. This was reasonable, accounting for difference in their MSSS values as well as the observed fact of gender specificity in associations of miRNA genetic variance with MS susceptibility.

Carriage of the MIR499A*C/T genotype and the biallelic combination (MIR499A*C/T + MIR196A2*C) were strongly associated with an MSSS value >3.5, *i.e.*, with a severe MS course. Of note is that the significance levels of these associations in women (*p*_corr_ = 2.23 × 10^−5^ and 4.43 × 10^−6^, correspondingly) were higher than in the entire group (*p*_corr_ = 0.0032 and 0.00078, correspondingly). If we consider the carriage of the MIR499A*C allele and the combination (MIR499A*C + MIR196A2*C), the observed significance levels substantially decrease in comparison with of the MIR499A*C/T genotype by itself and in combination with MIR196A2*C. Both combinations (MIR499A*C/T + MIR196A2*C) and (MIR499A*C + MIR196A2*C) satisfy the requirement of the minimal predisposing set of alleles in the entire group and in women. Association of identified genetic variants with MS severity was confirmed using the Mann-Whitney test: their carriers have significantly higher MSSS values (*p* = 0.028–0.0003) both among women and in the entire group. At the same time no associations with MS severity were found in men.

The observed ORs for miRNA genetic variance associations with MS severity are quite high. They lay in ranges from 1.61 to 2.12 in the entire group and from 2.21 to 3.23 in women. Such strong effect ranges are not common for complex diseases. The fact that the highest ORs and the lowest *p*-values in this study are obtained for a heterozygous genotype at *MIR499A* rs3746444 corresponds to the codominant genetic model.

rs3746444, located in pre-miR-499a, was previously found to be associated with several autoimmune diseases. Different alleles and genotypes of this gene were significantly associated with rheumatoid arthritis in Iranians [23], ulcerative colitis in Japanese [25], Behcet’s disease in Turks [26], and with a severe and more active phenotype of rheumatoid arthritis in Egyptian female patients [44]. Of note is that the most significant associations in the two latter cases were found for the C/T genotype, which reinforces the evidence for possible codominant interaction between *MIR499A* alleles.

rs11614913*Т in the *MIR196A2* gene was shown to increase mature miR-196a2 expression and to influence its targets binding [21]. This allele has been previously associated with severe clinical phenotype of colitis in Japanese patients [25] and with higher risk of Bechet’s disease in Chinese patients [45]. rs11614913 is also a cis-eQTL for *SMUG1* gene [46,47], which encodes a base excision repair enzyme, involved in checkpoint regulation of the cell cycle and DNA reparation.

In all cases significant associations with MS susceptibility and severity were identified in women but not in men. Furthermore, in most instances the significance levels of these associations in women are up to several orders higher than in more numerous entire groups including both men and women. Association of MIR223*T allele carriage with MS risk was the only exception, being more significant in the entire group than in women, but location of this gene on the X chromosome did not favor analysis of gender effects. Given the above, there is every reason to suppose that the studied microRNA variants show gender specificity and are not associated with MS risk and severity in men.

Such gender-dependent effects of polymorphisms of miRNA genes on disease susceptibility and clinical features may be explained by variations in endocrine profiles between men and women. It was previously shown that estrogen treatment influences on expression of studied here mir-146a, miR-196a2 and miR-223 genes [48,49]. Thus, estrogen-dependent difference in miRNA activity may modify the effect of polymorphisms on MS risk and severity and lead to gender specificity.

Using multi-locus association analysis, we observed in this study the moderate cumulative effect of *MIR223* and *MIR146A* genetic variants on MS susceptibility in women and the more strongly expressed cumulative effect of *MIR499A* and *MIR196A2* on MS course both in the entire group and in women. The joint contribution of two miRNA genes may arise from their additive effect or from possible modifying effect (epistasis) of the gene by the other gene. One way or another, observed interactions of miRNA genes may reflect their pairwise involvement in different pathways of the miRNA regulatory network.

## 4. Experimental Section

### 4.1. Patients and Controls

Five hundred sixty-one unrelated patients with bout-onset MS diagnosed according to the McDonald Criteria [50] were enrolled in the study (395 women, 166 men; mean age 38.7 ± 10.7 years). 464 patients suffered from RRMS and 97 from SPMS. Retrospectively collected clinical data also included age of MS onset, disease duration, EDSS value [38], and MSSS calculated according to the Global MSSS table [6] and measured before starting therapy.

The control group consisted of 441 volunteers with no sign of neurological disorders (mean age 44.4 ± 16 years). Among them there were 285 women and 149 men whereas for 7 individuals gender was unknown. All MS patients and healthy individuals lived in the Moscow region and self-reported as Russians. The study was approved by the local ethics committee, and written informed consent had been obtained from each patient.

### 4.2. Genotyping

Extraction of genomic DNA from whole or frozen blood samples was performed either with modified phenol-chloroform method [51] or using QIAamp DNA Blood Midi Kits from Qiagen (Hilden, Germany). Real-time PCR with pre-designed TaqMan kits from Life Technologies (Foster City, CA, USA) was used for genotyping of SNPs in miRNA genes: *MIR499A* rs3746444, *MIR223* rs1044165, *MIR196A2* rs11614913, *MIR146A* rs2910164.

### 4.3. Statistical Analysis

Observed genotype frequencies were compared by *χ*^2^ criteria (the significance level set at 0.05) with expected genotype proportions calculated according to the Hardy-Weinberg equilibrium. The Mann-Whitney test was used to evaluate the differences in MS clinical features in various groups of patients.

Alleles, genotypes and combinations of alleles of different loci, carriage of which was associated with MS risk and severity, along with the direction of each association, were identified using the APSampler algorithm [52]. The findings were then validated by standard statistical approaches: calculation of OR and 95% CI and the exact Fisher’s *p*-value (*p*_f_) using the tools included in the APSampler software [53]. In this case, we applied a one-sided Fisher’s test for the validation procedure, as the direction of association for each pattern to be validated was already known. We considered MS association with a single allele/genotype to be nominally significant, if *p*_f_ was <0.05, and with allelic combinations, if *p*_f_ was <0.01. In all cases we required that the 95% CI for OR did not cross 1. All genetic variants were considered to be significantly associated with a studied phenotype if their Bonferroni-corrected exact Fisher’s test *p*-values (*p*_corr_) were <0.05.

## 5. Conclusions

We found that SNPs in *MIR223* and *MIR146A* are involved in MS susceptibility, whereas SNPs in *MIR499A* and *MIR196A2* are involved in the MS course. Multi-locus association analysis has shown pairwise interactions of *MIR223* with *MIR146A* and *MIR499A* with *MIR196A2*. The distinct roles of the studied miRNA variants in MS risk and severity may indicate divergent regulatory networks to be involved in the development of MS and in formation of a severe clinical phenotype in patients. Significant associations of miRNA genes’ variants were gender-specific, having been identified in women but not in men. However, replication of the obtained results in individuals of the same and of other ancestries is the next necessary study stage.

## Supplementary Materials

Supplementary materials can be found at http://www.mdpi.com/1422-0067/16/08/20067/s1.
