# Supplementary material for: Variants of MicroRNA Genes: Gender-Specific Associations with Multiple Sclerosis Risk and Severity
Source: Int J Mol Sci. 2015 Aug 24;16(8):20067–81. doi: 10.3390/ijms160820067 (PMC4581341; doi:10.3390/ijms160820067)
Supplement: Supplementary file 1 [file ijms-16-20067-s001.pdf]

# Supplementary Information

**Table S1.** Comparison of observed miRNA genotype frequencies with expected frequences according to Hardy-Weinberg equilibrium.

**Healthy Controls**

| - | Gene SNP ID | Genotype | Observed No. of Patients | Observed Genotype Frequencies | Expected Genotype Frequencies | Expected No. of Patients | $\chi^2$ | *p*-Value |
|---|---|---|---|---|---|---|---|---|
| Entire group | *MIR146A* rs2910164 | C/C | 20 | 0.045 | 0.048 | 21 | | |
| | | C/G | 153 | 0.347 | 0.342 | 151 | 0.097 | 0.760 |
| | | G/G | 268 | 0.608 | 0.611 | 269 | | |
| | *MIR196A2* rs11614913 | C/C | 172 | 0.390 | 0.400 | 176 | | |
| | | C/T | 214 | 0.485 | 0.465 | 205 | 0.854 | 0.360 |
| | | T/T | 55 | 0.125 | 0.135 | 60 | | |
| | *MIR499A* rs3746444 | C/C | 13 | 0.029 | 0.034 | 15 | | |
| | | C/T | 138 | 0.313 | 0.302 | 133 | 0.503 | 0.480 |
| | | T/T | 290 | 0.658 | 0.663 | 293 | | |
| Women | *MIR146A* rs2910164 | C/C | 12 | 0.042 | 0.054 | 15 | | |
| | | C/G | 108 | 0.379 | 0.356 | 101 | 1.196 | 0.270 |
| | | G/G | 165 | 0.579 | 0.591 | 168 | | |
| | *MIR196A2* rs11614913 | C/C | 113 | 0.396 | 0.396 | 113 | | |
| | | C/T | 133 | 0.467 | 0.466 | 133 | 0.00044 | 0.99 |
| | | T/T | 39 | 0.137 | 0.137 | 39 | | |
| | *MIR499A* rs3746444 | C/C | 4 | 0.014 | 0.028 | 8 | | |
| | | C/T | 88 | 0.309 | 0.280 | 80 | 2.982 | 0.084 |
| | | T/T | 193 | 0.677 | 0.691 | 197 | | |
| | *MIR223* rs1044165 | C/C | 220 | 0.772 | 0.763 | 217 | | |
| | | C/T | 58 | 0.203 | 0.221 | 63 | 1.729 | 0.190 |
| | | T/T | 7 | 0.025 | 0.016 | 5 | | |

**MS Patients**

| - | Gene SNP ID | Genotype | Observed No. of Patients | Observed Genotype Frequencies | Expected Genotype Frequencies | Expected No. of Patients | $\chi^2$ | *p*-Value |
|---|---|---|---|---|---|---|---|---|
| Entire group | *MIR146A* rs2910164 | C/C | 30 | 0.053 | 0.043 | 24 | | |
| | | C/G | 172 | 0.307 | 0.328 | 184 | 2.402 | 0.12 |
| | | G/G | 359 | 0.64 | 0.630 | 353 | | |
| | *MIR196A2* rs11614913 | C/C | 220 | 0.392 | 0.391 | 219 | | |
| | | C/T | 262 | 0.467 | 0.468 | 263 | 0.0051 | 0.94 |
| | | T/T | 79 | 0.141 | 0.140 | 79 | | |
| | *MIR499A* rs3746444 | C/C | 21 | 0.037 | 0.038 | 21 | | |
| | | C/T | 176 | 0.314 | 0.313 | 175 | 0.0029 | 0.96 |
| | | T/T | 364 | 0.649 | 0.650 | 364 | | |
| Women | *MIR146A* rs2910164 | C/C | 15 | 0.038 | 0.036 | 14 | | |
| | | C/G | 121 | 0.306 | 0.309 | 122 | 0.034 | 0.85 |
| | | G/G | 259 | 0.656 | 0.654 | 259 | | |
| | *MIR196A2* rs11614913 | C/C | 158 | 0.400 | 0.398 | 157 | | |
| | | C/T | 182 | 0.461 | 0.466 | 184 | 0.050 | 0.82 |
| | | T/T | 55 | 0.139 | 0.137 | 54 | | |
| | *MIR499A* rs3746444 | C/C | 13 | 0.033 | 0.036 | 14 | | |
| | | C/T | 124 | 0.314 | 0.308 | 122 | 0.165 | 0.68 |
| | | T/T | 258 | 0.653 | 0.656 | 259 | | |
| | *MIR223* rs1044165 | C/C | 273 | 0.691 | 0.691 | 273 | | |
| | | C/T | 111 | 0.281 | 0.280 | 111 | 0.0051 | 0.94 |
| | | T/T | 11 | 0.028 | 0.028 | 11 | | |

**Table S1.** *Cont.*

| | Healthy Controls | | | | | | | | MS Patients | | | | | | | |
|---|---|---|---|---|---|---|---|---|---|---|---|---|---|---|---|---|
| **-** | **Gene SNP ID** | **Genotype** | **Observed No. of Patients** | **Observed Genotype Frequencies** | **Expected Genotype Frequencies** | **Expected No. of Patients** | **$\chi^2$** | ***p*-Value** | **-** | **Gene SNP ID** | **Genotype** | **Observed No. of Patients** | **Observed Genotype Frequencies** | **Expected Genotype Frequencies** | **Expected No. of Patients** | **$\chi^2$** | ***p*-Value** |
| Men | *MIR146A* rs2910164 | C/C | 8 | 0.054 | 0.039 | 6 | 1.241 | 0.270 | Men | *MIR146A* rs2910164 | C/C | 15 | 0.090 | 0.059 | 10 | 4.653 | **0.031** |
| | | C/G | 43 | 0.288 | 0.318 | 47 | | | | | C/G | 51 | 0.307 | 0.368 | 61 | | |
| | | G/G | 98 | 0.658 | 0.643 | 96 | | | | | G/G | 100 | 0.603 | 0.572 | 95 | | |
| | *MIR196A2* rs11614913 | C/C | 56 | 0.376 | 0.411 | 61 | 3.440 | 0.064 | | *MIR196A2* rs11614913 | C/C | 62 | 0.373 | 0.377 | 63 | 0.049 | 0.83 |
| | | C/T | 79 | 0.530 | 0.460 | 69 | | | | | C/T | 80 | 0.482 | 0.474 | 79 | | |
| | | T/T | 14 | 0.094 | 0.129 | 19 | | | | | T/T | 24 | 0.145 | 0.149 | 25 | | |
| | *MIR499A* rs3746444 | C/C | 8 | 0.054 | 0.049 | 7 | 0.108 | 0.740 | | *MIR499A* rs3746444 | C/C | 8 | 0.048 | 0.042 | 7 | 0.245 | 0.62 |
| | | C/T | 50 | 0.336 | 0.345 | 51 | | | | | C/T | 52 | 0.313 | 0.325 | 54 | | |
| | | T/T | 91 | 0.610 | 0.605 | 90 | | | | | T/T | 106 | 0.639 | 0.633 | 105 | | |

Significant *p*-values are in bold.

**Table S2.** Case-control comparison of genetic variants in miRNA genes with MS risk.

| Gene SNP ID | Carriage of Genotypes and Alleles | Carriers (%) MS Cases *n* = 561 | Carriers (%) Controls *n* = 441 | *p*-Value | OR (95% CI) | Carriers (%) MS Cases *n* = 395 | Carriers (%) Controls *n* = 285 | *p*-Value | OR (95% CI) | Carriers (%) MS Cases *n* = 166 | Carriers (%) Controls *n* = 149 | *p*-Value | OR (95% CI) |
|---|---|---|---|---|---|---|---|---|---|---|---|---|---|
| | | Without gender stratification | | | | Women | | | | Men | | | |
| *MIR146A* rs2910164 | C/C | 30 (5.3) | 20 (4.5) | 0.33 | 1.19 (0.67–2.12) | 15 (3.8) | 12 (4.2) | 0.47 | 0.90 (0.41–1.95) | 15 (9.0) | 8 (5.4) | 0.15 | 1.75 (0.72–4.26) |
| | C/G | 172 (30.7) | 153 (34.7) | 0.10 | 0.83 (0.64–1.09) | 121 (30.6) | 108 (37.9) | **0.030** | 0.72 (0.53–1.00) | 51 (30.7) | 43 (28.8) | 0.41 | 1.09 (0.67–1.77) |
| | G/G | 359 (64.0) | 268 (60.8) | 0.16 | 1.15 (0.89–1.48) | 259 (65.6) | 165 (57.9) | **0.025** | 1.39 (1.01–1.90) | 100 (60.3) | 98 (65.8) | 0.19 | 0.79 (0.50–1.25) |
| | C | 202 (36.0) | 173 (39.2) | 0.16 | 0.87 (0.67–1.13) | 136 (34.4) | 120 (42.1) | **0.025** | 0.72 (0.53–0.99) | 66 (39.8) | 51 (34.2) | 0.19 | 1.27 (0.80–2.01) |
| | G | 531 (94.7) | 421 (95.5) | 0.33 | 0.84 (0.47–1.50) | 380 (96.2) | 273 (95.8) | 0.47 | 1.11 (0.51–2.42) | 151 (91.0) | 141 (94.6) | 0.15 | 0.57 (0.23–1.39) |
| *MIR196A2* rs11614913 | C/C | 220 (39.2) | 172 (39.0) | 0.50 | 1.01 (0.78–1.30) | 158 (40.0) | 113 (39.6) | 0.50 | 1.01 (0.74–1.39) | 62 (37.3) | 56 (37.6) | 0.53 | 0.99 (0.63–1.56) |
| | C/T | 262 (46.7) | 214 (48.5) | 0.31 | 0.93 (0.72–1.19) | 182 (46.1) | 133 (46.7) | 0.47 | 0.98 (0.72–1.33) | 80 (48.2) | 79 (53.0) | 0.23 | 0.82 (0.53–1.28) |
| | T/T | 79 (14.1) | 55 (12.5) | 0.26 | 1.15 (0.80–1.66) | 55 (13.9) | 39 (13.7) | 0.51 | 1.02 (0.66–1.59) | 24 (14.5) | 14 (9.4) | 0.11 | 1.63 (0.81–3.28) |
| | C | 482 (86.0) | 386 (87.5) | 0.26 | 0.87 (0.60–1.26) | 340 (86.1) | 246 (86.3) | 0.51 | 0.98 (0.63–1.52) | 142 (85.5) | 135 (90.6) | 0.11 | 0.61 (0.30–1.24) |
| | T | 341 (60.8) | 269 (61.0) | 0.50 | 0.99 (0.77–1.28) | 237 (60.0) | 172 (60.4) | 0.50 | 0.99 (0.72–1.35) | 104 (62.7) | 93 (62.4) | 0.53 | 1.01 (0.64–1.60) |

**Table S2.** *Cont.*

| Gene SNP ID | Carriage of Genotypes and Alleles | Carriers (%) MS Cases *n* = 561 | Carriers (%) Controls *n* = 441 | *p*-Value | OR (95% CI) | Carriers (%) MS Cases *n* = 395 | Carriers (%) Controls *n* = 285 | *p*-Value | OR (95% CI) | Carriers (%) MS Cases *n* = 166 | Carriers (%) Controls *n* = 149 | *p*-Value | OR (95% CI) |
|---|---|---|---|---|---|---|---|---|---|---|---|---|---|
| | | Without gender stratification | | | | Women | | | | Men | | | |
| *MIR499A* rs3746444 | C/C | 21 (3.7) | 13 (2.9) | 0.24 | 1.38 (0.67–2.84) | 13 (3.3) | 4 (1.4) | 0.095 | 2.38 (0.77–7.38) | 8 (4.8) | 8 (5.4) | 0.51 | 0.89 (0.32–2.42) |
| | C/T | 176 (31.4) | 138 (31.3) | 0.52 | 1.00 (0.76–1.30) | 124 (31.4) | 88 (30.9) | 0.49 | 1.02 (0.73–1.42) | 52 (31.3) | 50 (33.6) | 0.41 | 0.92 (0.57–1.48) |
| | T/T | 364 (64.9) | 290 (65.8) | 0.40 | 0.96 (0.74–1.25) | 258 (65.3) | 193 (67.7) | 0.30 | 0.90 (0.65–1.25) | 106 (63.9) | 91 (61.0) | 0.38 | 1.11 (0.70–1.75) |
| | C | 197 (35.1) | 151 (34.2) | 0.40 | 1.04 (0.80–1.36) | 137 (34.7) | 92 (32.3) | 0.30 | 1.11 (0.80–1.53) | 60 (36.1) | 58 (38.9) | 0.38 | 0.90 (0.57–1.43) |
| | T | 540 (96.3) | 428 (97.1) | 0.24 | 0.72 (0.35–1.49) | 382 (96.7) | 281 (98.6) | 0.095 | 0.42 (0.14–1.30) | 158 (95.2) | 141 (94.6) | 0.51 | 1.13 (0.41–3.09) |
| *MIR223* rs1044165 | C/C | - | - | - | - | 273 (69.1) | 220 (77.2) | **0.012** | 0.66 (0.47–0.94) | - | - | - | - |
| | C/T | - | - | - | - | 111 (28.1) | 58 (20.3) | **0.013** | 1.53 (1.06–2.20) | - | - | - | - |
| | T/T | - | - | - | - | 11 (2.8) | 7 (2.5) | 0.50 | 1.14 (0.44–2.97) | - | - | - | - |
| | C | 517 (92.2) | 413 (93.7) | 0.22 | 0.80 (0.49–1.30) | 384 (97.2) | 278 (97.5) | 0.50 | 0.88 (0.34–2.30) | 133 (80.1) | 128 (85.9) | 0.11 | 0.66 (0.36–1.20) |
| | T | 155 (27.6) | 86 (19.5) | **0.0017** | 1.58 (1.17–2.13) | 122 (30.9) | 65 (22.8) | **0.012** | 1.51 (1.07–2.15) | 33 (19.9) | 21 (14.1) | 0.11 | 1.51 (0.83–2.75) |

Significant *p*-values are in bold.

**Table S3.** Case-control comparison of genetic variants in miRNA genes with MSSS.

| Gene SNP ID | Carriage of Genotypes and Alleles | Carriers (%) Without gender stratification MSSS > 3.5 n = 304 | MSSS ≤ 3.5 n = 242 | p-Value | OR (95% CI) | Carriers (%) Women MSSS > 3.5 n = 207 | MSSS ≤ 3.5 n = 181 | p-Value | OR (95% CI) | Carriers (%) Men MSSS > 3.5 n = 97 | MSSS ≤ 3.5 n = 61 | p-Value | OR (95% CI) |
|---|---|---|---|---|---|---|---|---|---|---|---|---|---|
| *MIR146A* rs2910164 | C/C | 15 (4.9) | 13 (5.4) | 0.48 | 0.91 (0.43–1.96) | 8 (3.9) | 7 (3.9) | 0.60 | 1.00 (0.36–2.81) | 7 (7.2) | 6 (9.8) | 0.38 | 0.71 (0.23–2.23) |
| | C/G | 91 (30.0) | 75 (31.0) | 0.43 | 0.95 (0.66–1.37) | 61 (29.4) | 57 (31.5) | 0.37 | 0.91 (0.59–1.40) | 30 (30.9) | 18 (29.5) | 0.50 | 1.07 (0.53–2.15) |
| | G/G | 198 (65.1) | 154 (63.6) | 0.39 | 1.07 (0.75–1.52) | 138 (66.7) | 117 (64.6) | 0.38 | 1.09 (0.72–1.67) | 60 (61.9) | 37 (60.7) | 0.51 | 1.05 (0.55–2.03) |
| | C | 106 (34.9) | 88 (36.4) | 0.39 | 0.94 (0.66–1.33) | 69 (33.3) | 64 (35.4) | 0.38 | 0.91 (0.60–1.39) | 37 (38.1) | 24 (39.3) | 0.51 | 0.95 (0.49–1.83) |
| | G | 289 (95.1) | 229 (94.6) | 0.48 | 1.09 (0.51–2.35) | 199 (96.1) | 174 (96.1) | 0.60 | 1.00 (0.36–2.82) | 90 (92.8) | 55 (90.2) | 0.38 | 1.40 (0.45–4.39) |
| *MIR196A2* rs11614913 | C/C | 110 (36.2) | 101 (41.7) | 0.11 | 0.79 (0.56–1.12) | 77 (37.2) | 78 (43.1) | 0.14 | 0.78 (0.52–1.18) | 33 (34.0) | 23 (37.7) | 0.38 | 0.85 (0.44–1.66) |
| | C/T | 153 (50.3) | 103 (42.6) | 0.043 | 1.37 (0.97–1.92) | 103 (49.8) | 76 (42.0) | 0.076 | 1.37 (0.92–2.04) | 50 (51.6) | 27 (44.3) | 0.23 | 1.34 (0.70–2.55) |
| | T/T | 41 (13.5) | 38 (15.7) | 0.27 | 0.84 (0.52–1.35) | 27 (13.0) | 27 (14.9) | 0.35 | 0.86 (0.48–1.52) | 14 (14.4) | 11 (18.0) | 0.35 | 0.77 (0.32–1.82) |
| | C | 263 (86.5) | 204 (84.3) | 0.27 | 1.19 (0.74–1.93) | 180 (87.0) | 154 (85.1) | 0.35 | 1.17 (0.66–2.08) | 83 (85.6) | 50 (82.0) | 0.35 | 1.30 (0.55–3.10) |
| | T | 194 (63.8) | 141 (58.2) | 0.11 | 1.26 (0.89–1.79) | 130 (62.8) | 103 (56.9) | 0.14 | 1.28 (0.85–1.92) | 64 (66.0) | 38 (62.3) | 0.38 | 1.17 (0.60–2.29) |

**Table S3.** *Cont.*

| Gene SNP ID | Carriage of Genotypes and Alleles | Carriers (%) | | *p*-Value | OR (95% CI) | Carriers (%) | | *p*-Value | OR (95% CI) | Carriers (%) | | *p*-Value | OR (95% CI) |
|---|---|---|---|---|---|---|---|---|---|---|---|---|---|
| | | MSSS > 3.5 | MSSS ≤ 3.5 | | | MSSS > 3.5 | MSSS ≤ 3.5 | | | MSSS > 3.5 | MSSS ≤ 3.5 | | |
| | | *n* = 304 | *n* = 242 | | | *n* = 207 | *n* = 181 | | | *n* = 97 | *n* = 61 | | |
| | | Without gender stratification | | | | Women | | | | Men | | | |
| *MIR499A* rs3746444 | C/C | 7 (2.3) | 11 (4.5) | 0.11 | 0.49 (0.19–1.30) | 3 (1.4) | 9 (5.0) | 0.043 | 0.28 (0.075–1.05) | 4 (4.1) | 2 (3.3) | 0.57 | 1.27 (0.23–7.15) |
| | C/T | 112 (36.8) | 58 (24.0) | **0.00081** | 1.85 (1.27–2.70) | 86 (41.6) | 37 (20.4) | **0.0000056** | 2.77 (1.76–4.36) | 26 (26.8) | 21 (34.4) | 0.20 | 0.70 (0.35–1.40) |
| | T/T | 185 (60.9) | 173 (71.5) | **0.0059** | 0.62 (0.43–0.89) | 118 (57.0) | 135 (74.6) | **0.00020** | 0.45 (0.29–0.70) | 67 (69.1) | 38 (62.3) | 0.24 | 1.35 (0.69–2.65) |
| | C | 119 (39.1) | 69 (28.5) | **0.0059** | 1.61 (1.12–2.32) | 89 (43.0) | 46 (25.4) | **0.00020** | 2.21 (1.44–3.41) | 30 (30.9) | 23 (37.8) | 0.24 | 0.74 (0.38–1.45) |
| | T | 297 (91.8) | 231 (95.5) | 0.11 | 2.02 (0.77–5.29) | 204 (98.6) | 172 (95.0) | 0.043 | 3.56 (0.95–13.35) | 93 (95.9) | 59 (96.7) | 0.57 | 0.79 (0.14–4.44) |
| *MIR223* rs1044165 | C/C | - | - | - | - | 140 (67.6) | 129 (71.3) | 0.25 | 0.84 (0.55–1.30) | - | - | - | - |
| | C/T | - | - | - | - | 62 (30.0) | 46 (25.4) | 0.19 | 1.25 (0.80–1.96) | - | - | - | - |
| | T/T | - | - | - | - | 5 (2.4) | 6 (3.3) | 0.41 | 0.72 (0.22–2.41) | - | - | - | - |
| | C | 283 (93.1) | 220 (91.0) | 0.22 | 1.35 (0.72–2.51) | 202 (97.6) | 175 (96.7) | 0.41 | 1.39 (0.42–4.62) | 81 (83.5) | 45 (73.8) | 0.10 | 1.80 (0.82–3.94) |
| | T | 83 (27.3) | 68 (28.1) | 0.46 | 0.96 (0.66–1.40) | 67 (32.4) | 52 (28.7) | 0.25 | 1.19 (0.77–1.83) | 16 (16.5) | 16 (26.2) | 0.10 | 0.56 (0.25–1.22) |

Significant *p*-values are in bold.
